# YouTube User Traffic to Paired Epilepsy Education Videos in English and Spanish: Comparative Study

**DOI:** 10.2196/56720

**Published:** 2025-03-13

**Authors:** Luna Kimahri Varela, Stephanie Horton, Ahmed Abdelmoity, Jean-Baptiste Le Pichon, Mark A Hoffman

**Affiliations:** 1 Children’s Mercy Hospital Kansas City, Children’s Mercy Research Institute, 2401 Gillham Road, Kansas City, MO, 64108, United States, 1 8169201931

**Keywords:** epilepsy, patient education, informatics, social media, biomedical research, social determinants of health, accessibility, engagement, comparative analysis, clinical videos, English, Spanish, neurological disorder, YouTube, bilingual, audience engagement, clinical knowledge

## Abstract

**Background:**

Effectively managing epilepsy in children necessitates the active engagement of parents, a factor that is reliant on their understanding of this neurological disorder. Widely available, high-quality, patient-focused, bilingual videos describing topics important for managing epilepsy are limited. YouTube Analytics is a helpful resource for gaining insights into how users of differing backgrounds consume video content.

**Objective:**

This study analyzes traffic to paired educational videos of English and Spanish versions of the same content. By examining the use patterns and preferences of individuals seeking information in different languages, we gained valuable insights into how language influences the use of clinical content.

**Methods:**

Physician experts created epilepsy management videos for the REACT (Reaching Out for Epilepsy in Adolescents and Children Through Telemedicine) YouTube channel about 17 subjects, with an English and Spanish version of each. The Children’s Mercy Kansas City neurology clinic incorporated these into the department’s educational process. YouTube Analytics enabled analysis of traffic patterns and video characteristics between September 2, 2021, and August 31, 2023.

**Results:**

The Spanish group had higher engagement and click-through rates. The English versions of all videos had 141,605 total impressions, while impressions for the Spanish versions totaled 156,027. The Spanish videos had 11,339 total views, while the English videos had 3366. The views per month were higher for the Spanish videos (mean 472, SD 292) compared to the English set (mean 140, SD 91; *P*<.001). The two groups also differed in search behavior and external traffic sources, with WhatsApp driving more traffic to the Spanish videos than the English versions (94 views compared to 1). The frequency of search terms used varied by language. For example, “tonic clonic” was the most frequent term (n=372) resulting in views for English videos, while “tipos de convulsiones” (types of convulsions) was the most common expression (n=798) resulting in views for Spanish videos. We noted increased monthly views for all videos after adding tags on YouTube. Before tagging, the mean number of views per month for the English-language group was 61 (SD 28), which increased to 220 (SD 53) post tagging. A similar trend can be observed in the Spanish-language group as well. Before tagging, the mean number of monthly views was 201 (SD 71), which increased to 743 (SD 144) after tagging.

**Conclusions:**

This study showed high traffic for Spanish video content related to epilepsy in a set of paired English/Spanish videos. This highlights the importance of bilingual health content and optimizing video content based on viewer preferences and search behavior. Understanding audience engagement patterns through YouTube Analytics can further enhance the dissemination of clinical video content to users seeking content in their primary language, and tagging videos can have a substantial impact on views.

## Introduction

### Video Health Content

Access to informative and reliable clinical content is crucial for managing complex clinical conditions, especially for individuals in rural areas or with limited access to specialist care [1]. Epilepsy, a common chronic condition, highlights the challenges faced by patients needing access to clinical specialists. Children and youth with epilepsy and their families need accurate and trustworthy information to manage their condition effectively [2].

Social media platforms, particularly YouTube, have become significant sources of information for many people. With billions of users monthly, YouTube has established its presence in over 100 countries worldwide and offers localized versions in 80 languages to serve a diverse global population [3]. However, poor-quality health videos on YouTube can be misleading, but they are still promoted via YouTube’s search algorithm that prioritizes popularity, relevance, and view history over content quality and accuracy [4]. This presents a significant challenge for individuals who seek health information without proper guidance or supervision, as they are increasingly exposed to unverified and partially misleading content. The consequences of such exposure can include the promotion of unhealthy habits and activities [5]. A 2023 study about video-sharing platforms revealed a strong negative correlation between high-quality content and popularity regarding videos providing accurate information on cancer and nutrition [6]. Out of 1200 videos, the most reliable videos on YouTube were from universities, professional organizations, and nonprofit physician groups, while unreliable videos included content related to treatments that scored low on the Journal of American Medical Association scale for quality medical content [7]. Analysis of video content using the DISCERN instrument yielded similar results for diabetes content [8]. High-quality health care educational resources developed by experts on widely accessible platforms such as YouTube offer an approach to mitigating these concerns. Through analytics available through YouTube, we can understand the factors influencing viewership and engagement with clinical videos.

### REACT

Through the REACT (Reaching Out for Epilepsy in Adolescents and Children Through Telemedicine) project, Children’s Mercy Kansas City has developed online educational resources for families and children and youth with epilepsy [9]. One of these resources includes online videos covering different aspects of managing epilepsy and seizures, such as general content about seizures, types of seizures, seizure precautions, alternative treatments, and frequently asked questions. The goal was to provide access to patients and their families, primarily those who spoke English or Spanish. This design provided an opportunity to compare the use of videos with the same clinical content. A preliminary analysis of YouTube Analytics traffic patterns for one of these videos (“What is an absence seizure?”) showed high use for the Spanish version [7]. This paper presents a comprehensive traffic analysis of the REACT YouTube channel, comparing the paired English and Spanish versions of otherwise identical content.

## Methods

### Epilepsy Video Development and Deployment

Videos for epilepsy-related topics prioritized by the clinical team were developed in English and Spanish. A multistep approach was taken when translating the videos from English to Spanish. First, the bilingual research team translated the scripts into Spanish. The scripts were reviewed by bilingual and culturally competent staff before recording the videos. The resulting videos were uploaded to the REACT YouTube channel. The videos for each topic are considered as episodes with two versions.

### YouTube Analytics

YouTube Analytics for the REACT channel were accessed to evaluate online traffic patterns to the video content [10]. No personal identifiers were available or sought from YouTube. We did not attempt to correlate patient-prescribed views with the traffic patterns evaluated in this work. GraphPad Prism 9.5.1 (GraphPad Software, Inc) was used to quantify video characteristics, with continuous variables presented as means with SDs and ranges.

Tags are descriptive keywords used on YouTube to improve the discoverability of a video. In August 2022, the study team added tags to each of the videos. To analyze the impact of these tags, total monthly views were separated into a pretagging period (September 2021 to August 2022) and a posttagging period (September 2022 to August 2023).

Impressions are the number of times a video’s thumbnail is presented to a user on YouTube. The click-through rate was provided by YouTube Advanced Analytics and represents the percentage of REACT channel thumbnails that were clicked by viewers.

### Ethical Considerations

All study data used were anonymous.

## Results

### Epilepsy Video Development and Deployment

Clinical experts developed video content for 17 topics related to treating epilepsy and seizures. The Children’s Mercy Kansas City neurology clinic adopted these videos as part of the patient departure educational process. Children and youth with epilepsy and their families were “prescribed” to view at least one video after each visit. The information and prescribed video were printed in the educational department and given to patients so they could scan a QR code that led them to a REDCapÒ survey. This survey included demographic information, a link to the prescribed YouTube video, and a pre- and postsurvey. Children and youth with epilepsy and families also had the option to view more than one video if desired. The first videos were uploaded to the REACT YouTube channel on August 7, 2020, and all videos were made publicly available by September 2, 2021 [11].

### YouTube Analytics

**Figure 1.**
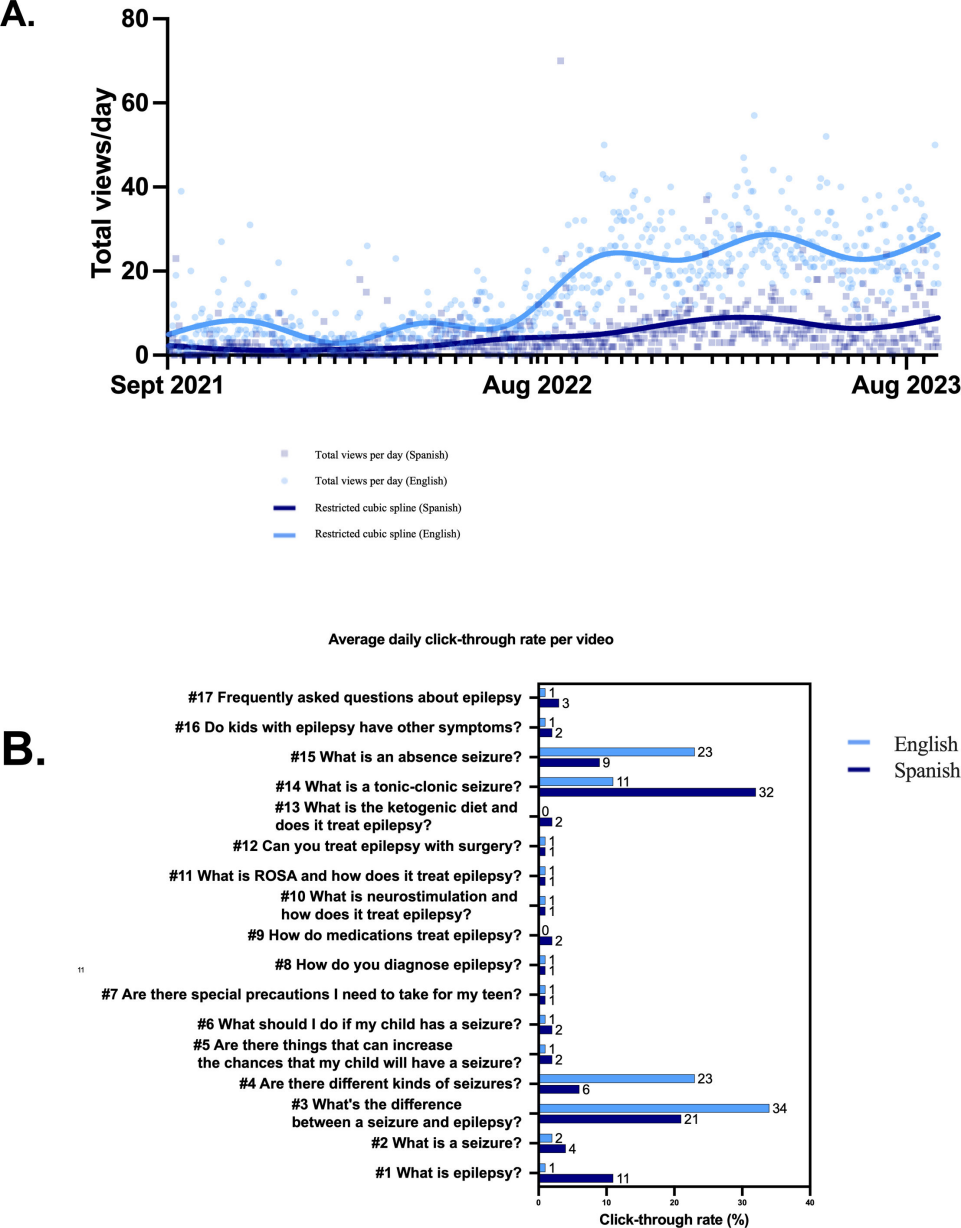
Daily views, by language, of epilepsy YouTube channel. (1A) The total daily views of the REACT (Reaching Out for Epilepsy in Adolescents and Children Through Telemedicine) YouTube channel from September 2, 2021, to August 31, 2023. The x-axis represents the total daily views, and a smoothing spline curve is included. (1B) Average daily click-through rate per video grouped by language. ROSA: robotic stereotactic assistance.

We evaluated traffic patterns between September 2, 2021, when all videos were available, and August 31, 2023. In the aggregate, views per day for the Spanish videos surpassed the English versions (Figure 1A). Compared to the English videos, traffic to the Spanish-language videos was significantly higher (*P*<.001) based on click-through rate (Figure 1B). Some episodes, such as 4 and 14, exhibited different click-through rates between the two languages.

**Figure 2.**
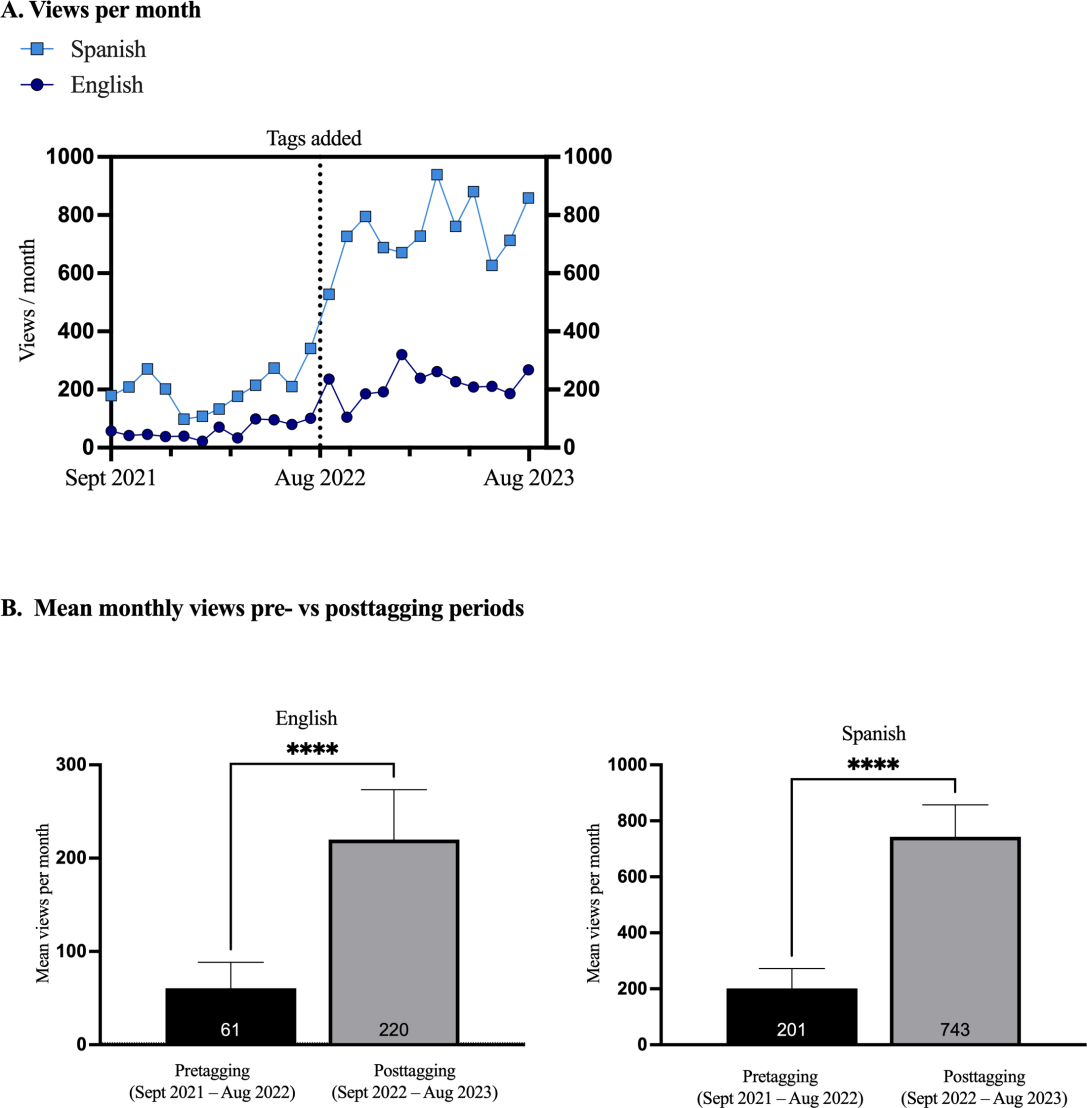
Impact of tagging: Total views per month grouped by language. (2A) Total views per month. Monthly views for all videos on the REACT (Reaching Out for Epilepsy in Adolescents and Children Through Telemedicine) YouTube channel were collected from September 2, 2021, to August 31, 2023, and grouped by language. The dotted line represents the addition of tags. Each data point indicates the total views per language group for that month. (2B) Mean monthly views pre- versus posttagging periods. Comparison of mean total monthly views before and after adding tags in August 2022 for English- and Spanish-language group videos. Paired *t* tests showed a significant difference in views. ****P<.001.

The addition of tags correlates with higher traffic (Figure 2A). The highest total monthly views for the English-language group of videos was 320 in January 2023, while the Spanish-language group of videos’ highest monthly views were 939 in March 2023. To further assess the impact of the tags, the total monthly views were divided into two periods: pretagging period (September 2021 to August 2022) and posttagging period (September 2022 to August 2023). In the pretagging period, the views per month for the English-language group increased after tagging (pretagging mean 61, SD 28; posttagging mean 220, SD 53). A similar trend was observed in the Spanish-language group as well (pretagging mean 201, SD 71; posttagging mean 743, SD 114). Both the English- and Spanish-language videos showed significant increases in traffic after the addition of tags (*P*<.001; Figure 2B).

**Figure 3.**
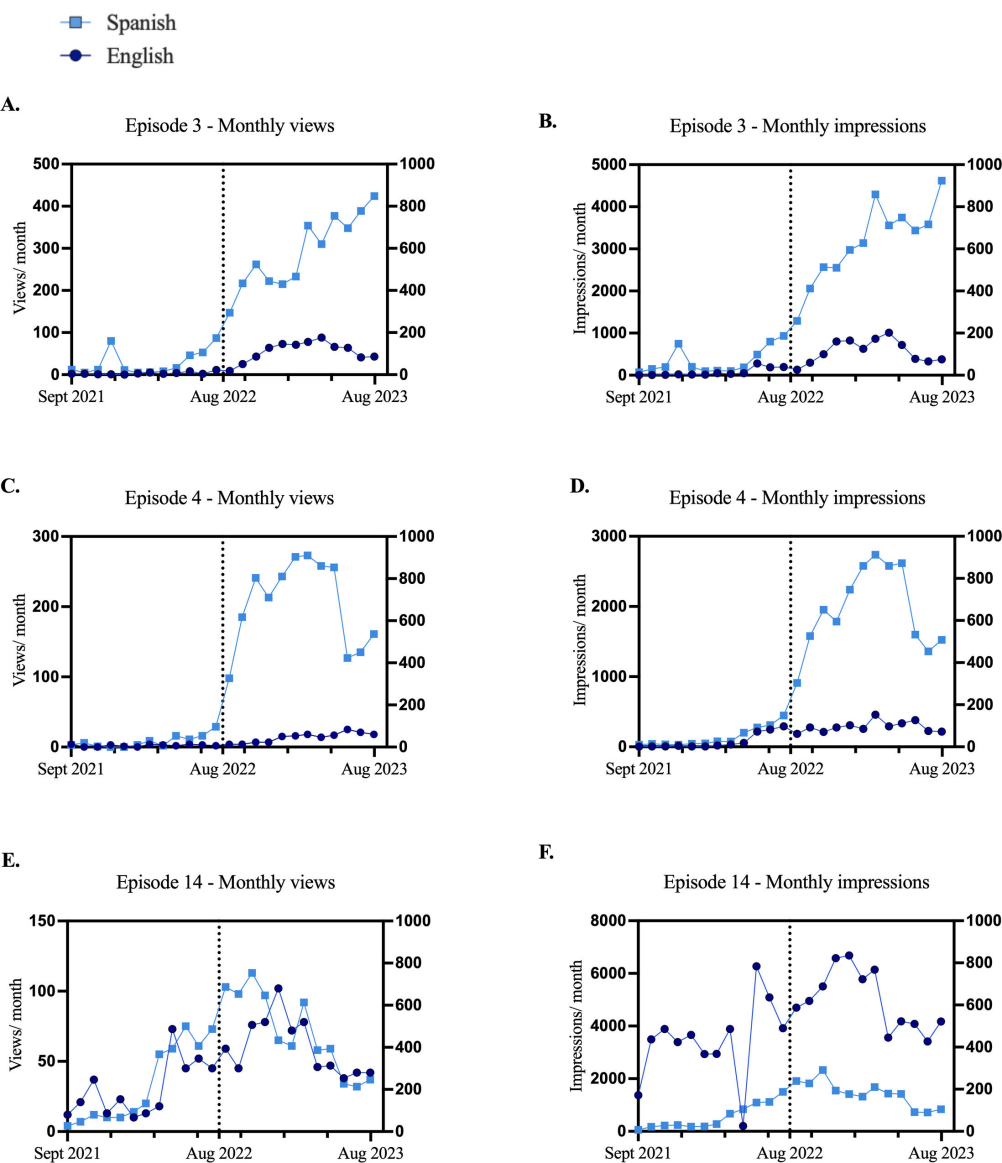
Monthly impressions and views for videos 3, 4, and 14. (3A, C, and E) Monthly views per month across time for videos 3, 4, and 14 from September 2021 to August 2023. (3B, D, and F) Monthly impressions across time for videos 3, 4, and 14.

Traffic to individual episodes demonstrated differing patterns. The Spanish versions of videos 3 and 4 showed a strong association between impressions and views (Figure 3A-D). For video 14, impressions and views were similar for the Spanish version, but for the English version, impressions increased earlier (Figure 3E-F). In the English category, video 14 had the highest impressions (n=106,936) and views (n=1087; Table 1). In the Spanish category, video 15 had the highest impressions (n=49,039), followed closely by video 3 (n=41,892). However, video 3 had more views (n=3839) than video 14.

**Table 1.** Impressions, click-through rate, and views for each epilepsy video.

	Video title		Total impressions, n		Click-through rate		Total views, n	
	English title	Spanish title	English	Spanish	English	Spanish	English	Spanish
1	What is epilepsy?	¿Que es la epilepsia?	454	806	4	3	352	106
2	What is a seizure?	¿Qué es una convulsión?	718	3523	4	5	144	255
3	What’s the difference between a seizure and epilepsy?	¿Cuál es la diferencia entre la epilepsia y una convulsión?	7727	41,892	7	7	706	3839
4	Are there different kinds of seizures?	¿Hay diferentes tipos de convulsiones?	4327	25,084	2	8	192	2557
5	Are there things that can increase the chances that my child will have a seizure?	¿Hay cosas que pueden aumentar las posibilidades de que tenga una convulsión?	341	902	5	4	51	66
6	What should I do if my child has a seizure?	¿Qué debo hacer si mi hijo tiene una convulsión?	1434	781	1	5	73	75
7	Are there special precautions I need to take for my teen?	¿Se deben tomar precauciones si mi hijo adolescente tiene epilepsia?	276	945	3	6	35	80
8	How do you diagnose epilepsy?	¿Cómo se diagnostica la epilepsia?	318	1519	3	5	33	111
9	How do medications treat epilepsy?	¿Cómo tratan los medicamentos la epilepsia?	1894	543	2	2	62	25
10	What is neurostimulation and how does it treat epilepsy?	¿Qué es la neuroestimulación y cómo trata la epilepsia?	355	2241	7	5	47	131
11	What is ROSA^a^ and how does it treat epilepsy?	¿Qué es ROSA y como trata la epilepsia?	326	606	4	4	25	35
12	Can you treat epilepsy with surgery?	¿Se puede tratar la epilepsia con cirugía?	214	1481	4	4	22	74
13	What is the ketogenic diet and does it treat epilepsy?	¿Qué es la dieta Ketogénica?	1106	439	4	4	79	26
14	What is a tonic-clonic seizure?	¿Cómo cuido a mi hijo con convulsiones tónico´-clónicas?	106,936	23,727	1	4	1087	1249
15	What is an absence seizure?	¿Qué es una crisis de ausencia?	13,319	49,039	2	4	304	2562
16	Do kids with epilepsy have other symptoms?	Otros sintomas de epilepsia	950	1968	2	4	65	115
17	Frequently Asked Questions about Epilepsy	Nuestra experta responde a sus preguntas frecuentes	910	531	6	4	89	33
Totals	—^b^	—	141,605	156,027	1	6	3366	11,339

a ROSA: robotic stereotactic assistance.

b Not applicable.

While the location of the viewer was only available for 2096 (14.3%) of the 14,705 views, we observed that countries with predominantly non–English-speaking populations tended to have higher view counts reported by YouTube than primarily English-speaking countries. Users in the United States generated the most views (n=884) for the Spanish language, followed by Mexico (n=859) and Argentina (n=110; Multimedia Appendix 1).

The results from the analysis of YouTube search terms used to find English and Spanish videos are presented in Table 2. The top searches that resulted in views for English videos were “tonic,” “clonic,” or “tonic-clonic.” The term “absence seizure” also generated strong interest, resulting in 118 views. Spanish videos had more views for the comparable search term “crisis de ausencia” or “absence seizure,” with 527 views. In comparison, the terms “convulsiones tonico clonico” or “tonic clonic seizures” only garnered 118 views. The search term “tipos de convulsiones” or “types of seizures” resulted in the most views for the Spanish-language group, with 798 views. These findings suggest differences in viewers’ search behavior when seeking content in different languages on YouTube.

**Table 2.** Most frequent YouTube search terms leading users to REACT (Reaching Out for Epilepsy in Adolescents and Children Through Telemedicine) content.

Search terms	Views, n
English language	
“tonic clonic” or “clonic” or tonic”	372
“Absence Seizure”	64
“what is a seizure”	17
“difference between seizure and epilepsy”	16
“seizure”	15
“types of seizures”	10
“epilepsy seizure”	8
“different types of seizures”	7
“difference between epilepsy and seizure”	5
“what is epilepsy”	5
Spanish language	
“tipos de convulsiones” (types of seizures)	798
“crisis de ausencia” (absence seizures)	527
“convulsiones tonico clonicas” (tonic-clonic seizures)	118
“que es una convulsión” (what is a seizure)	43
“convulsiones” (seizures)	40
“epilepsia” (epilepsy)	31
“que es la epilepsia” (what is epilepsy)	25
“como es una convulsión” (how is a seizure)	13
“convulsiones en adultos” (seizure’s in adults)	13
“convulsiones y epilepsia” (seizures and epilepsy)	13

Table 3 presents a comprehensive list of external traffic sources used to find video content on the REACT YouTube channel. English viewers predominantly accessed the content through the Children’s Mercy website, with 443 views, compared to the Spanish count of 151. Spanish-language content was embedded on an epilepsy education website maintained by the government of Chile and generated 121 views. Some of the Spanish-language video views originated from a Spanish-language occupational health education consultancy group’s website. Spanish-language viewers relied more heavily on WhatsApp as their preferred means of finding content, with 94 views compared to only 1 among English-language viewers.

**Table 3.** Traffic sources used by viewers to find REACT (Reaching Out for Epilepsy in Adolescents and Children Through Telemedicine) content.

Traffic source	Views, n	
	English language	Spanish language
Children’s Mercy website	443	161
Google	201	73
YouTube	42	35
Yahoo	2	3
Instagram	1	0
WhatsApp	1	94
bing.com	1	0
live.com	1	5
telegram.org	1	0
revistachilenadeepilepsia.cl	0	121
WhatsApp Business	0	2
precoinprevencion.com	0	10
Facebook	0	12

Much of the traffic to the REACT channel originated from sources outside the YouTube platform. These include internet searches, direct URL entries, bookmarks, or embedded links. External traffic sources accounted for 38,172 (12.8%) of the 297,632 impressions. However, despite the relatively lower number of impressions, these external sources, especially WhatsApp, contributed significantly to the overall viewership. In contrast, videos directly delivered by YouTube accounted for 259,460 (87.2%) impressions. Further analysis examined views by internal versus external sources, grouped by language. The Spanish-language groups contributed 8697 views internally and 2642 views externally to the YouTube platform, while the English videos generated 2508 views from within YouTube and 858 from external sources.

We investigated the impact of YouTube algorithmic suggestions on driving views to REACT content. These suggestions were presented as pop-ups after a video was completed or while users were browsing the website. Within the English group, we observed that videos discussing atonic seizures, tonic-clonic seizures, and the differences between seizures and epilepsy received the highest number of views, with 61, 31, and 14 views, respectively (Multimedia Appendix 2). The Spanish videos addressing convulsions and absence seizures garnered the most attention from these algorithmic recommendations, with 146 and 96 views, respectively.

## Discussion

YouTube reaches a larger audience than videos delivered through self-publication to isolated websites, for example, a university website [12]. This study evaluated the viewership of paired Spanish and English educational videos on the YouTube platform and noted substantial traffic to the Spanish-language versions relative to most of the English versions. One notable aspect of our work is the access to detailed analytics for videos with identical clinical content in English and Spanish, a distinguishing feature from other studies evaluating YouTube content for chronic disease [13]. The availability of such analytics provides valuable insights into the use patterns for clinical video content, allowing for informed content design and development strategies. YouTube analytics has substantial, yet underused, value to the developers of clinical content. While the primary intended use of YouTube metrics is to monitor and optimize ad views, we demonstrate that there is utility to these metrics for health education. By leveraging data-driven approaches, the quality and relevance of clinical content can be improved. This analysis enables the promotion of health equity by tailoring content and using search engine optimization features such as tagging to increase visibility.

Our analysis demonstrates a distinct difference in audience engagement between English- and Spanish-language videos providing the same content. Engagement is quantified by the number of views a video generates as well as other metrics, including view time, which we did not evaluate in this study. Overall, the Spanish-language group consistently exhibited higher levels of engagement, as evidenced by increased views and impressions. The modes of interaction differed; for example, Spanish speakers were more likely to use WhatsApp to access the content, consistent with the popularity of this messaging app among Spanish-speaking groups [14,15]. This finding highlights the importance of developing bilingual clinical content to meet the needs of diverse populations.

Furthermore, our study explored the correlation between impressions and views per month, providing insights into variations in monthly engagement across different videos.

The videos with the highest viewing rates were related to the clinical appearance of a seizure (“What’s the difference between a seizure and epilepsy?” “Are there different kinds of seizures?” “What is a tonic-clonic seizure?” “What is an absence seizure?”). This is in line with the public misconceptions of what a seizure looks like [16] and further emphasizes the importance of education regarding the many possible phenomenology of epileptic seizures [17].

While data about audience demographics was limited, our analysis showed that users in countries with predominantly non–English-speaking populations tended to have higher reported view counts of the Spanish versions of a video than users in primarily English-speaking countries. This suggests a global demand for clinical content in languages other than English, highlighting the need for bilingual or multilingual approaches to reach diverse audiences. The search behavior of viewers also differed between languages, as reflected in the top search terms leading to views for English and Spanish videos. Understanding these differences can inform content creators about the keywords and topics that resonate with their audiences, enabling them to optimize their content accordingly. For example, search terms most often leading users to a video can be added as tags.

External traffic sources (non-YouTube) significantly drove viewership. This highlights the importance of leveraging external platforms, such as websites and social media, to promote and distribute clinical video content posted on YouTube. WhatsApp was a frequent source of traffic to the Spanish videos, consistent with common use of this app in non–English-speaking countries [18]. Tailoring strategies to effectively reach and engage different target audiences based on language preferences and behaviors is crucial for maximizing the impact of clinical content dissemination. Our findings also demonstrate that the implementation of tags significantly improved the visibility and accessibility of the videos, leading to a substantial increase in total monthly views. This is consistent with previous research that has shown the effectiveness of tags in enhancing the discoverability of content on YouTube [19].

Finally, our analysis of suggestions generated by the YouTube platform showed variations in viewer preferences between the English and Spanish groups. This underscores the influence of recommendations in directing viewers toward specific content related to seizures and epilepsy. Content creators should consider these recommendations to increase viewership and engagement when developing and promoting their videos.

This study has known limitations. We focused solely on YouTube to evaluate clinical video viewership, and we acknowledge that other platforms may exhibit different use patterns and levels of audience engagement. The analysis was limited to English and Spanish videos, excluding other languages that may generate significant viewership. User privacy settings influence whether their location is visible in analytics, limiting access to granular geographic data and other demographic data (race, gender, age). Furthermore, the study did not explore the long-term effects of video viewership on health outcomes or behavior change. Lastly, we did not consider the influence of external factors, such as marketing or promotion efforts, on video viewership.

We demonstrated the effectiveness of paired educational videos in reaching a global audience well beyond the intended focus of the in-clinic recommendations. Techniques such as tags improve the discoverability and viewership of clinical videos on YouTube. The availability of detailed analytics provides valuable insights into audience engagement and use patterns, enabling informed content design and development strategies. Bilingual or multilingual approaches are essential for reaching diverse populations and promoting health equity. Leveraging external (outside of the YouTube platform) traffic sources and optimizing video content based on search behavior and viewer preferences can further enhance the impact of clinical video dissemination, for example, top YouTube searches that resulted in views related to the differences between types of seizures. The development of paired videos enabled us to perform side-by-side comparisons of traffic patterns and video characteristics on a specific health topic. Through this work, we elucidated practices to improve clinical content available on the YouTube platform for future health content creators seeking to support non–English-speaking users.

## Supplementary material

10.2196/56720 Multimedia Appendix 1 Total view count by country.

10.2196/56720 Multimedia Appendix 2 Top 10 traffic sources to REACT (Reaching Out for Epilepsy in Adolescents and Children Through Telemedicine) from other YouTube content grouped by language.

## Abbreviations

REACT: Reaching Out for Epilepsy in Adolescents and Children Through Telemedicine
