# Supplementary material for: YouTube User Traffic to Paired Epilepsy Education Videos in English and Spanish: Comparative Study
Source: JMIR Form Res. 2025 Mar 13;9:e56720. doi: 10.2196/56720 (PMC11924967; doi:10.2196/56720)
Supplement: Multimedia Appendix 1 [file formative-v9-e56720-s001.docx]

Multimedia Appendix 1: **Total View Count by Country**

| **Country** | **Total Views** |
| --- | --- |
| **United States** | 884 |
| **Mexico** | 859 |
| **Argentina** | 110 |
| **Columbia** | 99 |
| **Peru** | 33 |
| **Dominican Republic** | 18 |
| **South Africa** | 17 |
| **Nicaragua** | 13 |
| **Venezuela** | 12 |
| **Australia** | 11 |
| **Bolivia** | 10 |
| **El Salvador** | 10 |
| **Great Britain** | 10 |
| **India** | 10 |
| **Chile** | >10 |
