# Supplementary material for: YouTube User Traffic to Paired Epilepsy Education Videos in English and Spanish: Comparative Study
Source: JMIR Form Res. 2025 Mar 13;9:e56720. doi: 10.2196/56720 (PMC11924967; doi:10.2196/56720)
Supplement: Multimedia Appendix 2 [file formative-v9-e56720-s002.docx]

Multimedia Appendix 2: Top 10 Traffic Sources to REACT from other YouTube Content grouped by language.

| **English Language Videos that Resulted in Views** | **Views** | **Spanish Language Videos that Resulted in Views (English title)** | **Views** |
| --- | --- | --- | --- |
| *Atonic Head Drops after Tonic Seizure* | 61 | *Convulsiones: qué son, causas y tipos (Convulsions: what are they, causes and symptoms)* | 146 |
| *Seizures (Epilepsy) Nursing NCLEX: Tonic-Clonic, Generalized, Focal, Symptoms* | 31 | *Cómo tratar las CRISIS DE AUSENCIA (How to treat an absence seizure)* | 96 |
| *What's the Difference Between a Seizure and Epilepsy?* | 14 | *Cómo identificar las CRISIS DE AUSENCIA (How to identify an absence seizure)* | 69 |
| *Atonic Drop Seizure\|T-Tiny* | 10 | *Aprende sobre las diferencias entre LA EPILEPSIA y LAS CONVULSIONES✔👍Enfermedad - Síntoma (Learn the differences between epilepsy and a seizure)* | 62 |
| *Is there a difference between a "seizure disorder" and epilepsy? (Chad Carlson, MD)* | 9 | *017. Crisis de Ausencia (Absence Seizures)* | 61 |
| *What are Tonic-Clonic Seizures?* | 8 | *Tipos de convulsiones (Types of convulsions)* | 44 |
| *Types of Seizures from epilepsy.com* | 8 | *EPILEPSIA Y CONVULSIONES COMO NUNCA TE LO HAN EXPLICADO* | 39 |
| *Understanding the difference between Epilepsy and Seizures \| Dr. Harirama K Acharya* | 6 | *Convulsiones Tonico Clonico (Tonic-Clonic Convulsiones)* | 26 |
| *Difference Between Seizure And Convulsion? \|\| Seizure \|\| Convulsion \|\| Dr M B* | 5 | *¿Es lo mismo epilepsia y convulsiones? #shorts (Are Epilepsy and Convulsiones the same?)* | 22 |
| *Generalized Tonic Clonic Seizures* | 4 | *Dr. Javier Chapa: Convulsiones vs Epilepsia (Convulsions vs Epilepsy)* | 22 |
